# Functional and Gait Assessment in Children and Adolescents Affected by Friedreich’s Ataxia: A One-Year Longitudinal Study

**DOI:** 10.1371/journal.pone.0162463

**Published:** 2016-09-06

**Authors:** Gessica Vasco, Simone Gazzellini, Maurizio Petrarca, Maria Luisa Lispi, Alessandra Pisano, Marco Zazza, Gessica Della Bella, Enrico Castelli, Enrico Bertini

**Affiliations:** 1 Movement Analysis and Robotics Laboratory (MARLab), Neurorehabilitation Unit, Department of Neurosciences, IRCCS Bambino Gesù Children’s Hospital, Rome, Italy; 2 Unit of Neuromuscular and Neurodegenerative Disorders, Department of Neurosciences, IRCCS Bambino Gesù Children's Hospital, Rome, Italy; 3 Neurology Unit, Department of Neurosciences, Bambino Gesù Children's Hospital, IRCCS, Rome, Italy; Sant Joan de Déu Children's Hospital, SPAIN

## Abstract

Friedreich’s ataxia is the most common autosomal recessive form of neurodegenerative ataxia. We present a longitudinal study on the gait pattern of children and adolescents affected by Friedreich’s ataxia using Gait Analysis and the Scale for the Assessment and Rating of Ataxia (SARA). We assessed the spectrum of changes over 12 months of the gait characteristics and the relationship between clinical and instrumental evaluations. We enrolled 11 genetically confirmed patients affected by Friedreich’s ataxia in this study together with 13 normally developing age-matched subjects. Eight patients completed a 12-month follow-up under the same protocol. By comparing the gait parameters of Friedreich’s ataxia with the control group, we found significant differences for some relevant indexes. In particular, the increased knee and ankle extension in stance revealed a peculiar biomechanical pattern, which correlated reliably with SARA Total, Gait and Sitting scores. The knee pattern showed its consistency also at the follow-up: Knee extension increased from 6.8±3.5° to -0.5±3.7° and was significantly correlated with the SARA total score. This feature anticipated the loss of the locomotor function in two patients. In conclusion, our findings demonstrate that the selective and segmental analysis of kinetic/kinematic features of ataxic gait, in particular the behavior of the knee, provides sensitive measures to detect specific longitudinal and functional alterations, more than the SARA scale, which however has proved to be a reliable and practical assessment tool. Functional outcomes measures integrated by instrumental evaluation increase their sensitivity, reliability and suitability for the follow-up of the disease progression and for the application in clinical trials and in rehabilitative programs.

## Introduction

Friedreich’s ataxia (FRDA) is the most common autosomal recessive form of ataxia that affects approximately 1 in 50,000 Caucasians[1]. FRDA is caused by autosomal recessive GAA trinucleotide expansion in the first intron of the FXN gene on the proximal long arm of chromosome 9, which interferes with frataxin transcription. Moreover 1–3% of the patients are heterozygosus for the GAA expansions on one allele and a point mutation or deletion on the other allele of FXN[2]. Typical age at onset occurs around or even before puberty, although showing a very large variability even between siblings[3]. Progressive gait ataxia, dysmetria, dysdiadocokinesia, muscular weakness, sensory loss, areflexia are typical clinical features of the disease. In addition to neurodegenerative symptoms, there is a multiple systemic involvement that includes cardiomyopathy, scoliosis, diabetes mellitus, foot deformities, abnormalities of eye movements. FRDA patients are wheelchair bound by a disease duration of 15.5 years (range 3–44) with a shortened life expectancy [4,5].

One of the challenges for therapeutic clinical trials in FRDA is the development of outcome measures, which should have good reliability, validity, reproducibility and sensitivity to change. Nowadays, more than in the past, the longitudinal studies of FRDA disease are important to analyze disease progression and to improve the accuracy of prognosis but mostly to prove the adequacy and the sensitivity to change of the outcome measures[6]. An accurate knowledge of the disease natural history is in fact a critical assumption upon which a clinical trial has to be designed.

The Scale for the Assessment and Rating of Ataxia (SARA)[7] for functional assessments has been recognized as the most sensitive scale in a longitudinal analysis of FRDA patients in comparison with ICARS and FARS[6]. SARA has been considered the best choice for its high construct validity, its good effect size and for its compact structure. Sensitivity to longitudinal change of SARA was evaluated in adult FRDA subjects by Marelli and coll.[8] Furthermore, although there are no validated scales for childhood, SARA is reliably applicable to children beyond the age of 10 years and proved to be more suitable for long-term quantitative ataxia assessment from child- to adulthood in comparison to ICARS and BARS[9]. However, the complexity of the neurological phenotype of FRDA due to the intricate interplay between cerebellar degeneration, somatosensory loss and muscle atrophy leads to explore the specific functional and gait changes over time more deeply and with the use of sensitive and objective measures.

Moreover, as well-known in the literature[10–13], the high intra-subject variability of all gait measures is the peculiar and distinctive aspect of ataxic gait that needs an accurate and exhaustive evaluation. However, in FRDA only a few studies have investigated the walking pattern by means of an objective gait analysis. These studies reported a good relationship between gait parameters and the clinical status of disease [12–16].

Unfortunately, these studies have focused only on spatiotemporal parameters (e.g. velocity, step length, single-double support %, etc.) of the gait[13] or in the adult population of FRDA[15]. Other studies compared gait parameters among ataxic patients with the heterogeneous etiology of ataxia[12,17]. Furthermore, only few prospective natural history studies are reported in the literature and all the studies have used simply functional rating scales to describe the progression rate of impairment and disability in FRDA[18–20].

To the best of our knowledge, no longitudinal study on FRDA patients employing gait analysis has been described so far, although this methodology of functional evaluation of gait progression proved to be a useful tool in longitudinal studies both on normal children during growth[21] and on affected children with Cerebral Palsy[22].

In the present longitudinal study, we report changes in a one-year time frame of the gait analysis and SARA in a cohort of children and adolescents affected by FRDA. The goal of this study was threefold: First, to investigate analytically the FRDA gait pattern, through an accurate measurement of kinematic and kinetic data in comparison with healthy controls. Second, to assess the spectrum of changes over 12 months in the individual measures and the correlation between clinical and objective assessment. Third, to identify among the redundant indices of gait analysis which parameters better reflect the core gait pattern of FRDA in terms of sensitivity to change in a longitudinal evaluation and in relation to the functional disease status. Our hypothesis is that functional outcomes measures integrated by instrumental evaluation may increase their sensitivity, reliability and suitability for the follow-up of the disease progression and for the application in clinical trials and in rehabilitative programs.

## Materials and Methods

### Study Design

This is a longitudinally observational cohort study with two assessments in a one-year follow-up.

### Subjects and Clinical assessment

Eleven genetically confirmed FRDA patients (age range 6.9–17.8; mean±SD 13.4±3 years; 8 females) were enrolled in this study. Patients were age-matched with 13 typically developing children and adolescents (age range 5.5–14;mean±SD10.3±3; 7females). Table 1 shows the clinical and functional characteristics of our cohort. Nine participants were able to walk independently, 2 patients needed mild walking aids (cans). All the patients were taking idebenone (10 mg/kg/day) for more than 6 months at the time of the study[23–26]. All the patients were able to complete the entire protocol in an outpatient setting. No patient had visual impairment except for slight abnormalities of eye movements (fixation instability, square wave jerks).

**Table 1:** Patients clinical and demographic overview.

Subjects	GAA smaller	GAA longer	Disease duration (y)	Onset age	BMI	Age (y)	Sara Tot	Age (y) FU	Sara Tot FU
F	780	1115	3	6.2	17.8	9.2	9.5	10.7	10
F	930	1066	7	7.4	19.2	14.4	15.5	15.4	18
M	780	780	6	8.9	18.6	14.8	10	16.5	13
M	600	750	6	7.9	14.4	13.9	14	15.2	19
F	715	900	7	7.7	23.6	14.7	9	16.0	10
F	633	633	6	6.6	21	12.6	21.5	14.1	29.5
F	900	1000	8	9.9	19.2	17.8	24.5		
M	805	1064	5	9.2	18.1	14.2	9.5		
F	264	1347	5	9.8	17.3	14.7	8	15.3	8.5
F	682	848	1	6	18.2	7.0	5	8.7	10.5
F	1014	1347	3	5.4	14.3	8.4	9	9.1	11

Legend: FU, follow up.

All the patients underwent a comprehensive assessment consisting in a complete history and a neurological evaluation. The disease status of all the patients was rated by the same neurologist specialized in ataxia by using the SARA.

Ten patients were evaluated again after a one-year follow-up (mean 15.2 months): two of them lost ambulation during the period between the two sessions. One patient dropped out of the follow-up. Therefore, at the follow-up ten patients were evaluated with the SARA scale, and eight of them were able to execute the gait analysis.

All the participants and their parents signed an informed consent before starting the evaluation session. The procedure was approved by the Ethics Committee of “Bambino Gesù” Children’s Hospital.

### Gait evaluation: Equipment

Standardized gait analysis was conducted by eight-camera motion capture system (Vicon MX, UK) and two force plates (AMTI, Or6). The sampling rates was set at 200 Hz for motion capture system and at 2 kHz using the two force plates (AMTI, or-6, US). The two force plates were hidden in the middle portion of a 10 meters walkway. Assessments were video recorded to assist clinical interpretation of data. After some familiarization trials, when the patients had reached their own self-selected barefoot speed, a specific starting position was selected in order to achieve the whole foot landing on the force plate avoiding any further verbal instructions. 33 markers were located on anatomical landmarks of the subjects as indicated by the Plug-in-Gait protocol in order to reconstruct a full body kinematic and kinetic model. Fifteen body segments were modelled. Kinematic and kinetic temporal series were normalized to the stride duration. Walking velocity and step length were normalized to leg length. Kinetic data were normalized to subject’s weight.

**Fig 1:**
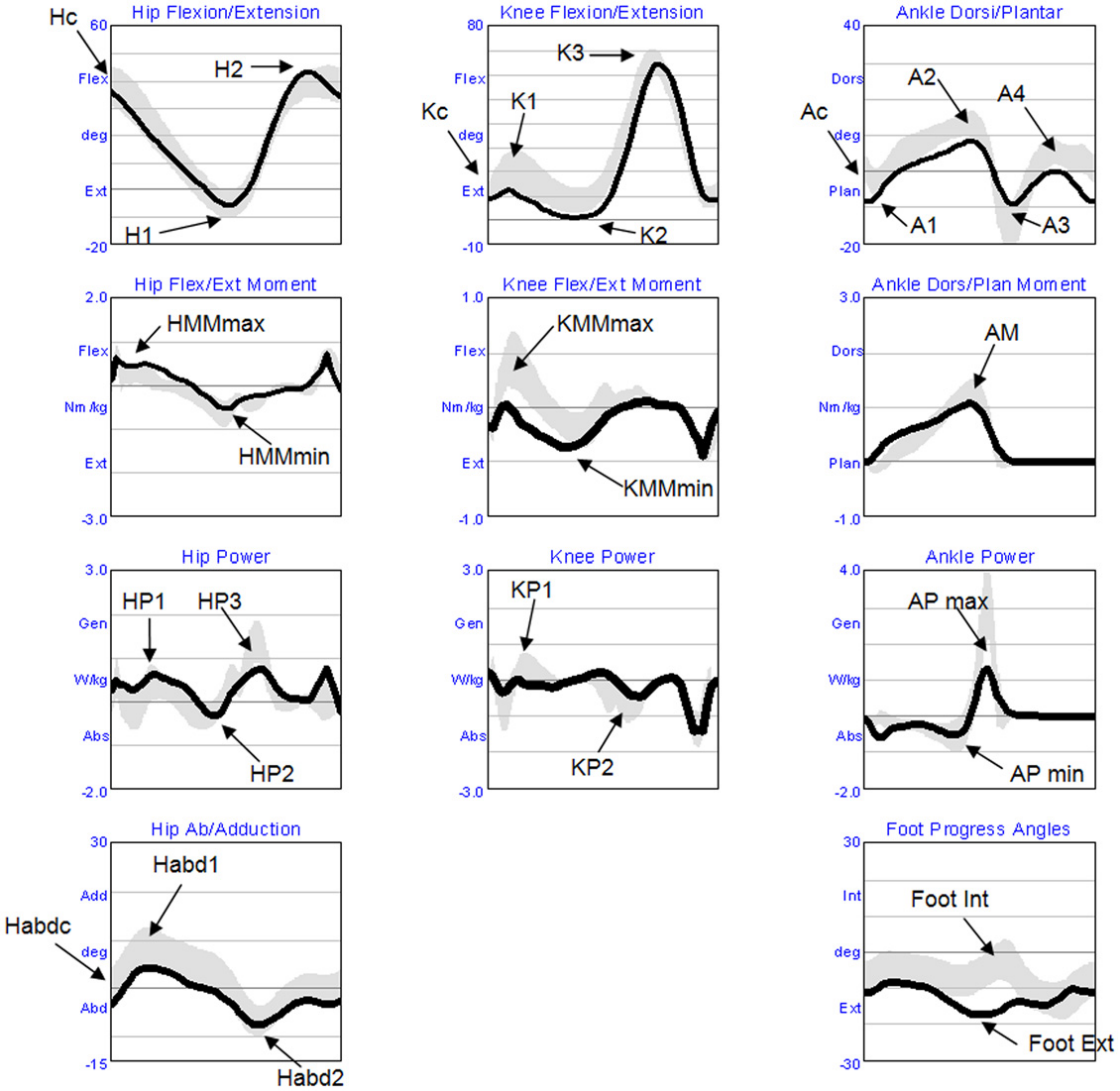
Kinetic/Kinematics parameters. The figure shows the time series of the variables examined in the paper. In the horizontal axis is represented the time normalized respect to the duration of the gait cycle (from the hell contact to the floor to the ipsilateral succeeding hell contact). The arrows indicate the peak values here studied. The black line represents the mean values of the baseline of the patient with FRDA, while the gray band represents the standard deviation of the control group. In the first row are represented in degree the flexion and extension rotation of the hip, the knee and the ankle, from left to right respectively. In the second and third rows are represented joints moment and power, respectively, normalized respect to body wheight. In the four row is represented the hip ab-adduction and the angle of the internal end external rotation of the foot.

The following list of variables was selected to detect the main gait strategies adopted by the patients and compared with a cohort of age-matched healthy subjects. In particular, we considered: walking velocity, step and stride length, stride time, step width, single support (SS) and double support (DS) percentage, lateral displacement of the centre of mass (COM), hip angle at initial contact (Hc), hip maximum extension (H1), hip maximum flexion (H2), hip abduction in stance (Habdc and Habd1) and in swing (Habd2), knee angle at initial contact (Kc), knee flexion during load response (K1), knee extension in late stance (K2), knee flexion during swing (K3), ankle angle at initial contact (Ac), plantar flexion during load response (A1), dorsal flexion in late stance (A2), plantar flexion in early swing (A3), dorsal flexion in swing (A4), peaks of foot intra-rotation and extra-rotation (Foot-Int, Foot-Ext), maximum and minimum hip moments (HMmax, HMmin) and knee moments during stance (KMmax, KMmin), maximum ankle dorsal moment in stance (AM), hip (HP1, HP2, HP3), knee (KP1, KP2), and ankle (APmax, APmin) maximum power generation and absorption during stance (see Fig 1).

### Statistical analysis

The t-Student’s test with Bonferroni correction was applied to identify statistical significance between patients with FRDA and controls. The values of step length, stride length and step width have been normalized for participant’s height. Repeated measures t-Student’s tests were executed to verify statistical differences between the baseline and the follow-up values of the patients. T-Student’s tests were similarly applied to evaluate statistical differences on the standard deviations between FRDA and controls and for the patients between baseline and follow-up. The differences between standard deviations were assumed as an index of variability in the motor pattern. Correlational analyses were also executed among the variables of the study: demographic, genetic, clinical variables and gait analysis parameters. Statistical analyses were conducted using the software IBM-SPSS 20.

## Results

### Baseline analyses

By comparing the gait parameters of FRDA and control group, we found significant differences for several indexes, as reported in Table 2. Bonferroni correction for multiple comparisons were applied and α = 0.0013 has been assumed as threshold for significance (α = 0.05 divided by 37 comparisons). The patients showed a significant reduction of walking velocity (0.9 vs. 1.2 m/s, FRDA vs. controls: t_(81)_ = -6.6, p <.0013) with a shorter step (0.36 vs 0.41 m: t_(103)_ = -7, p <.0013) and stride length (0.71 vs 0.82 m: t_(107)_ = -7.8, p <.0013) and an increase of the stride time (1.3 vs 1 s: t_(130)_ = -8.8, p <.0013). The maximal lateral displacement of the COM were double than controls (77.8 vs 34 cm: t_(34)_ = 5, p <.0013), and the % of double support was increased (24.8 vs 17.7: t_(66)_ = 4.5, p <.0013).

**Table 2:** T-test results on average values and standard deviations of kinetic/kinematic parameters of Gait Analysis: FRDA (1^st^ assessment) vs Controls.

	Friedreich			Controls			t-Student (Mean)			t-Student (St_Dev)		
	mean	DS	N	mean	DS	N	t	df	p	t	df	p
COM (cm)	77.8	38	28	34	12.8	37	5	34	<.0013			
Hc (°)	38.2	4	62	40.6	2.4	82	-1.7	142	n.s.			
H1 (°)	-3.3	2.2	62	-3.4	1.8	83	0.1	143	n.s.			
H2 (°)	46.7	3.1	62	43.5	2	83	2.6	143	n.s.			
Kc(°)	12.8	3.4	62	12.5	2.5	82	0.3	142	n.s.			
K1 (°)	17.8	3.2	62	23.6	3.5	83	-3.3	115	<.0013			
K2 (°)	4.4	2.7	62	9.4	2.2	83	-5.5	143	<.0013			
K3 (°)	70.7	3.5	62	67.8	2.8	83	3.2	143	<.0013			
Ac (°)	-6.2	3.5	62	0.84	2.5	82	-5.9	79	<.0013			
A1 (°)	-7.2	3	62	-3	1.9	83	-4.3	86	<.0013			
A2 (°)	11	4.6	62	14	2.4	83	-3.3	91	n.s. (.0014)	3.9	22	<.0013
A3 (°)	-14.1	4.5	62	-16	4.9	83	1.3	143	n.s.			
A4 (°)	2.4	4.8	62	5.9	2.3	83	-3.5	78	<.0013			
Velocity (m/s)	0.9	0.07	62	1.2	0.04	83	-6.6	81	<.0013			
StepLength (m)	0.36	0.03	62	0.41	0.02	83	-7	103	<.0013			
Stride Length (m)	0.71	0.03	62	0.82	0.03	83	-7.8	107	<.0013			
Stride Time (s)	1.3	0.8	62	1	0.09	83	3.5	62	<.0013			
StepWidth (m)	0.13	0.05	62	0.09	0.01	83	2.7	65	n.s.			
AM (Nm/kg)	1.15	0.1	57	1.30	0.1	75	-3.6	130	<.0013			
APmax (W/kg)	2.1	0.4	57	3.3	0.5	75	-8.7	130	<.0013			
APmin (W/kg)	-0.6	0.3	57	-0.7	0.2	75	1.5	130	n.s.			
Foot-Ext (°)	-17.2	7.5	62	-14.2	4.2	83	-2.1	86	n.s.			
Foot-Int (°)	1.3	6.4	62	0.6	4.1	83	0.6	95	n.s.			
HMmax (Nm/kg)	0.8	0.2	56	0.5	0.1	75	5.5	129	<.0013			
HMmin (Nm/kg)	-0.6	0.1	57	-0.6	0.1	75	0.6	130	n.s.			
KMmax (Nm/kg)	0.3	0.2	56	0.5	0.1	75	-3.8	129	<.0013			
KMmin (Nm/kg)	-0.4	0.1	57	-0.2	0.1	75	-5.6	76	<.0013			
HP1 (W/kg)	1.2	0.3	56	0.7	0.2	75	4.2	96	<.0013			
HP2 (W/kg)	-0.5	0.3	57	-0.3	0.2	75	-2.5	130	n.s.			
HP3 (W/kg)	1.3	0.3	57	1.5	0.3	75	-1	88	n.s.			
KP1 (W/kg)	0.5	0.3	56	0.6	0.2	75	-1.3	129	n.s.			
KP2 (W/kg)	-0.9	0.3	57	-0.1	0.2	75	1.9	99	n.s.			
Habdc (°)	-0.6	3.5	62	0.3	2.8	82	-1.2	107	n.s.			
Habd1 (°)	9.2	3	62	9	2.5	82	0.3	142	n.s.			
Habd2 (°)	-7	2.6	62	-7.6	2.5	82	1.1	142	n.s.			
SS (%)	38.5	3.8	62	41	1.3	77	-2.7	67	n.s.	8.5	22	<.0013
DS (%)	24.8	4.4	62	17.7	1.4	77	4.5	66	<.0013			

hip angle at initial contact (Hc), hip maximum extension (H1), hip maximum flexion (H2), knee angle at initial contact (Kc), knee flexion during load response (K1), knee extension in late stance (K2), knee flexion during swing (K3), ankle angle at initial contact (Ac), plantar flexion during load response (A1), dorsal flexion in late stance (A2), plantar flexion in early swing (A3), dorsal flexion in swing (A4), maximum ankle dorsal moment in stance (AM), ankle maximum power generation and absorption during stance (APmax, APmin), peaks of foot intra-rotation and extra-rotation (Foot-Int, Foot-Ext), maximum and minimum hip moments (HMmax, HMmin), maximum and minimum knee moments during stance (KMmax, KMmin), hip maximum power generation and absorption during stance (HP1, HP2, HP3), maximum power generation and absorption during stance (KP1, KP2), hip abduction in stance at initial contact (Habdc), in middle stance (Habd1) and in swing (Habd2), single support duration in percentage SS(%) and double support duration in percentage DS(%). See also Fig 1.

As far as the kinematic parameters were concerned, we observed a significant different behavioral pattern of the ankle and knee, in particular we found that Ac, A1, and A2 were significantly reduced in FRDA in the mean values (all p values <.0013, see Table 2), meaning increased plantar flexion. Also K1, K2 were reduced, whereas K3 was increased (all p <.0013) in FRDA in comparison to the healthy subjects. The analysis of kinetics showed significant differences in the moment AM, KMmax, KMmin and HMmax (all p <.0013) and in the power of AP max (p <.0013) and HP1 (p <.0013).

We also conducted the analysis of the differences between the standard deviations calculated for the patients with FRDA and controls in order to evaluate the previously described increase of gait variability in patients with FRDA. The results in Table 2 showed increasing variability in FRDA vs Controls in the maximum ankle plantar flexion during stance (A2; t_(22)_ = 3.9, p <.0013), and in single support percentage (SS; t_(22)_ = 8.5, p <.0013).

### Longitudinal analyses

Since the presence of multiple comparisons, Bonferroni correction were also applied and the threshold for significance has been assumed as α = 0.0013. Among all the significant parameters, only K1 (t_(7)_ = 6.5, p<.0013) and K2 (t_(7)_ = 8.4, p <.0013) were confirmed as statistically changed during the time of the follow-up. The differences at the SARA scale did not reach the corrected threshold for significance.

The standard deviations evidenced a stable behavior with no variation in follow-up.

**Fig 2:**
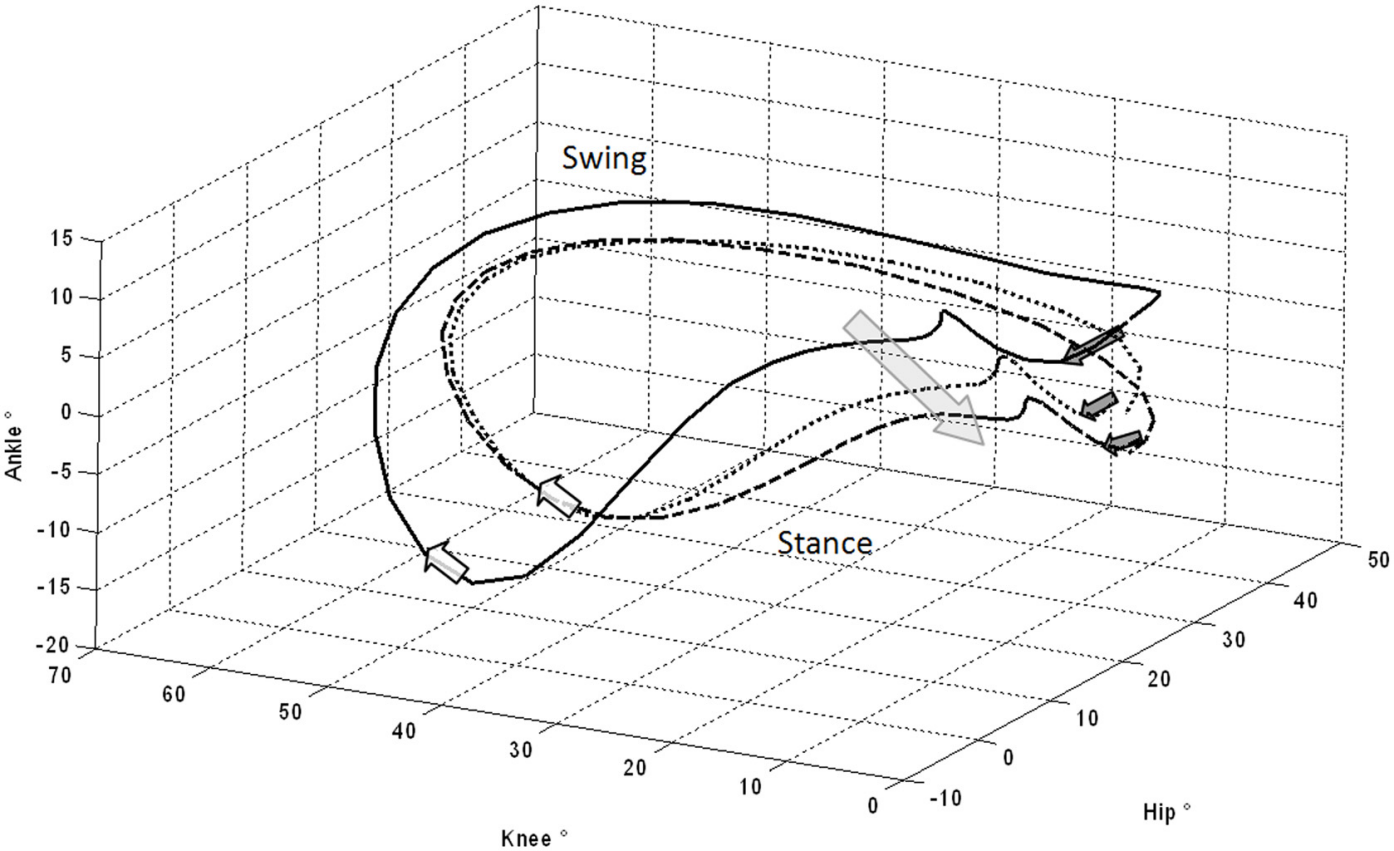
Ankle, hip and knee rotational co-variation during a complete gait cycle. The figure depicts the angles of hip, knee and ankle during the gait cycle on 3D plot, that is, the angle values on vertical axes of Fig 1 for hip, knee and ankle was bring back, respectively on x, y and z axes, in this figure. This figure represents the lower limb joint co-variation during the gait cycle. Solid line represents the mean values of controls, dotted line the baseline of FRDA, and dashed line the FRDA follow-up. Dark gray arrows indicate the foot landing, that is, the gait cycle start. White arrows indicate the beginning of the swing phase. The large light gray arrow represents the gait pattern progressive deterioration towards ankle and knee extension moving from the dotted to the dashed curves in patients with FRDA (see S1 Fig for a 3D animation of the graph).

For completeness of information longitudinal analysis without multiple comparison correction are also reported. The results demonstrated worsening of SARA scores: Total score (from 12.6 to 15.9; t_(9)_ = -4.2, p <.01); Gait (from 3 to 3.7; t_(9)_ = -2.7, p <.05); Stance (from 2.7 to 3.1; t_(9)_ = -2.4, p <.05); and Sitting (from 0.7 to 1.3; t_(9)_ = -3.7, p <.01). Analysis of kinematic data showed an increase of the knee shift towards extension during stance, as evidenced by the variation of Kc (from 14.4 to 8.2; t_(7)_ = 4.2, p <.01), K1 (from 21.2 to 11.4; t_(7)_ = 6.5, p <.001) and K2 (from 6.8 to -0.5; t_(7)_ = 8.4, p <.001). Moreover, the ankle showed similar behavior with a shift towards the plantar flexion evidenced by the variation of Ac (from -3.9 to -8.6; t_(7)_ = 2.4, p <.05), A1 (from -4.9 to -8.7; t_(7)_ = 3.4, p <.05) and A2 (from 12.7 to 10.1; t_(7)_ = 3.4, p <.05). In Fig 2 we illustrate this above mentioned statistical difference of gait pattern with a graphical representation of the co-variation plots of hip, knee and ankle angles. This graph shows clearly this difference of intra-limb coordination in comparison with controls, which became even more evident after one year follow-up (see S1 Fig for a 3D rotation of the hip-knee-ankle plot).

Kinetic data analysis evidenced decreased knee maximum extensor moment during early stance KMmax (from 0.38 to 0.12; t_(7)_ = 3.1, p <.05) and increased maximum flexor moment in late stance KMmin (from -0.3 to -0.5; t_(7)_ = 3.3, p <.05). The hip power generation in pre-swing phase HP3 (from 1.5 to 1.1; t_(7)_ = 3.3, p <.05) decreased as well.

Because of the great deal of variables in the correlation analysis and for sake of clarity, the full correlation matrix is reported in S1 Table. To sum up, we found a significant inverse correlation between velocity, percentage of the single support and SARA total score and all SARA-subscales (i.e. gait, stance, sitting, finger chase, nose to finger, heel-shin, all p values <.05). The stride time correlated directly and step and stride length correlated inversely with SARA total score and all the sub-scales (all p <.05). No correlation between GAA repeat size, disease duration and spatiotemporal parameters (walking velocity, step and stride length, stride time, step width, single support and double support %) were found. Conversely, we found a good correlation between the peaks of knee (Kc e K1) and GAA longer size and with disease duration (all p <.05). COM lateral displacement was negatively correlated to Habdc, Hp1, HMmax (all p <.05). SARA total score was inversely correlated with H2, Kc-2, Ac- A2 (all p <.05).

## Discussion

This study is a comprehensive analysis of the gait in a specific cohort of children and adolescents affected by FRDA, who were followed longitudinally for a period of 12 months. We analyzed the gait pattern in comparison with controls in terms of kinetic/kinematic characteristics and also in terms of variability of the measures. We analyzed the relationship between gait parameters and clinical and disease characteristics. Furthermore, we analyzed the spectrum of changes of all these parameters over a one-year follow-up.

### Baseline Analysis

It is known that several studies have focused on the description of the gait pattern of ataxic patients. The results of these previous studies were not always homogenous, ranging from deviations of spatiotemporal parameters and kinematic features[16,27,28] to normal values in other reports[27]. Some authors found gait alterations only in severe ataxia[29]. Contrasting findings are maybe due to the heterogeneity of the populations examined in these studies. However, if we restrict the search to the studies focusing on gait analysis of FRDA population, we find a more consistent pattern.

In agreement with previous works[12,13,15,16], our cohort of patient affected by FRDA showed slower velocity, shorter and wider step, increased stride time, more pronounced lateral displacement (COM), increased extra-rotation of the angle of foot progression and increased double support duration than healthy subjects.

### Biomechanical and Sensory Motor Explanation

The reduced walking speed and the seeking of an increased base of support is a clear and well-known compensatory strategy to prevent the loss of balance. Indeed typical stumbling and unsteady ataxic gait reflects the poor ability to maintain balance in subjects affected by FRDA, but reflects the impairments of intra-limb coordination as well, as shown by Ilg and coll[10,11]. Actually, FRDA pathophysiology is a mixed sensory and cerebellar ataxia resulting from spinocerebellar degeneration, peripheral sensory and optic neuropathy, cerebellar and vestibular pathology with a specific involvement of deep cerebellar nuclei (dentate, emboliform, globose and fastigial nucleus)[30,31]. The resulting walking pattern is the product of a complex interaction between cerebellar dysfunction and sensory loss, compromising balance control and multi-joint coordination.

The pattern of natural walking of our cohort of children and adolescent with FRDA in terms of kinematic and kinetic profiles showed a peculiar biomechanical strategy of gait characterized by a tendency to increase the ankle and knee extension during the stance phase. It seems that patients develop a compensation strategy characterized by knee joint stiffening in order to reduce the impairment in intra-limb coordination but also to compensate the disordered perception and peripheral neuropathy affecting the selective articulatory control. In other words, patients with FRDA lose the fine tuning of the knee joint, and as a consequence the lower limb is stabilized through knee hyper-extension during the stance phase. Longitudinal analysis showed a clear tendency to reiterate the use of such solution. The reduction of the control on intra-limb coordination could also be recognized from the increase of knee flexion in the swing phase that is followed by the increase of hip flexion. It is known, in fact, that the increase of knee flexion during swing produces the increase of hip flexion as a biomechanical consequence and not as a direct muscular control on the movement[32]. The increase in knee flexion facilitates the foot clearance thus avoiding undesired falls.

The reduction of the sensibility and of the selective muscular control produces increased exploitation of the biomechanical properties. Indeed, as shown in the correlation analysis provided in the S1 Table, the mean peaks of the knee were strongly correlated with stride time and velocity, but also with the moment and power of the ankle. This means that the strategy adopted determined a well-defined biomechanical configuration characterized by increased plantar flexion of the ankle at the initial phase of the contact, increased extension of the knee, reduction of the stride time and consequently reduction of the speed, reduction of the extensor moment of the knee and reduction of ankle power.

The kinematic aspect of intralimb co-variation, clearly identifiable in Fig 2, was sensitive to change in the follow-up and able to measure the progression of the disease in our FRDA cohort. Moreover this biomechanical configuration appears consistently correlated with the status of the disease, as evaluated with the SARA scale.

### Analysis on Variability

We also analyzed the variability of the gait parameters, which is a feature that has been widely recognized as particularly characteristic of ataxia[16]. The high gait variability of the patients with FRDA reflects both the deficit of balance control and the reduced a synergy of multi-joint movements[33]. In our clinical cohort we found significant higher variability compared to controls in terms of standard deviations of ankle maximum dorsiflexion and duration of the single support phase, and a tendency to significance (significant differences only without Bonferroni correction) of some parameters such as lateral displacement of the COM, ankle joint excursion and foot progression angle. These measures are already reported as a safety strategy in order to control the balance and to prevent accidental falls. It is worthy to note that this feature influenced the possibility to exert any anticipatory or predictive strategy in gait control[34]. The values of standard deviation were stable at the longitudinal analysis.

### Correlation Analysis

Taking into account the mean values of spatiotemporal parameters (velocity 0.9 m/sec, stride length 0.71 m, stride time 1.3 sec) and also the kinetic and kinematic features of our cohort, we argue that the patients were adopting a peculiar biomechanical safety strategy to prevent loss of balance and accidental falls. Consistently, we found a significant inverse correlation between velocity, percentage of the single support and SARA total score and all the analyzed SARA subscales (i.e. gait, stance, sitting, finger chase, nose to finger, heel-shin). The stride time correlated directly and step and stride length correlated inversely with SARA total score and all the subscales. This is partially in line with what was found in the study by Milne and coll. where the spatiotemporal gait parameters were highly correlated with clinical scales (FARS and 25FWT)[15]. In contrast with the already mentioned authors, we did not find any correlation between GAA repeat size, disease duration and spatiotemporal parameters. Conversely, we found a good correlation between peaks of knee (Kc e K1) and GAA longer size and with disease duration. COM lateral displacement was negatively correlated to Habd1, Hp1, HMmax. These data are consistent with the strategy of stabilizing the pelvis and the base of support in order to restrict the COM oscillation.

### Longitudinal Analysis

The t-Student’s test with Bonferroni correction highlighted the deterioration of the knee extension during the stance phase of the gait. As shown in Fig 2, the ankle-knee-hip covariation pattern of FRDA worsened between baseline and the follow-up evaluation. This feature anticipated the loss of the locomotor function in two patients. In this pediatric cohort of FRDA patients, the knee hyperextension was the only sensitive parameter for changes in one-year. However, at the follow-up, the patients with FRDA showed a clear clinical worsening, underlined also by the average changes of several parameters and SARA scores. These changes were significant applying the t-Student’s test without correction suggesting that in this particular case the Bonferroni correction could expose to the risk of false negative rejections (type II error). In fact, the analyses without correction showed a significant worsening of the SARA score (3.3± 2.5 mean SARA score variation points). Such SARA score annual variation is quite different from that reported in a previous study by Marelli and coll.[8], where a mean SARA score variation of 1.36 ± 2.3 points/year was described. However, this could be mainly due to different reasons: The first is that our cohort was composed of patients with early disease onset and faster worsening. The second reason is that our sample size was small, particularly at the follow-up. Further studies with larger sample size and longer follow-up will verify if this peculiar aspect of ataxic gait can be a predictive feature of locomotor status: In particular able to discriminate between independent walker and who need assistive device.

### Limitations

In our study, we analyzed only the self-selected speed of walking and this may represent a limitation because previous studies have demonstrated that in cerebellar ataxia a higher variability occurred only during slow walking and not during medium and fast speed[35,36]. However, the aim of our study was primarily to explore a possible common biomechanical characteristicof the gait in a specific cohort of ataxic patients. The selection of preferred walking mode is, in our experience, very representative of the skills and needs of daily living.

Although our study analyzed a limited sample size with a variable time of follow up, this is to our knowledge, the first work that reports a longitudinal natural history of gait in children and adolescent affected by FRDA using objective and exhaustive parameters of gait.

## Conclusions

The mean peaks of ankle and knee joint during stance demonstrated: 1. to be correlated with SARA scores; 2. to be sensitive to discriminate between FRDA and controls (p<0.001); 3. the knee peak was the most sensitive parameter for longitudinal changes in our cohort (p<0.0013). The peculiar co-variation between ankle and knee could be explained both by the sensorial and control deficit, which is distinctive in FRDA patients. In the longitudinal analysis, the knee hyperextension proved to be more sensitive to change respect to the other gait parameters and to SARA scale as well. Moreover, despite the main spatial and temporal parameters of gait analysis significantly correlated with SARA scores, these parameters proved not to be sensitive enough in longitudinal observation.

Finally, our findings demonstrate that the selective and segmental analysis of kinetic/kinematic features of ataxic gait provides sensitive measures to detect specific longitudinal and functional alterations, more than the SARA scale. Moreover such kinetic/kinematic analysis is useful not only in monitoring the motor function, but also in defining the compensatory walking strategy and pointing out individualized rehabilitative programs. SARA scale, in comparison with the parameters of gait analysis, represents a practical tool with a good sensitivity to changes also.

## Supporting Information

S1 Fig 3D Fig of animation of ankle, hip and knee rotational co-variation. (WMV)

S1 Table Correlation matrix FRDA 1^st^ assessment. * The correlation is significant at level 0.05 (2-code). **The correlation is significant at level 0.01 (2-code).
(XLS)
